# A Systematic Review and Meta-Analysis of Moderate-to-Vigorous Physical Activity Levels in Children and Adolescents With and Without ASD in Inclusive Schools

**DOI:** 10.3389/fped.2021.726942

**Published:** 2021-10-13

**Authors:** Ru Li, Xiao Liang, Yujuan Zhou, Zhanbing Ren

**Affiliations:** ^1^The Faculty of Physical Education, Shenzhen University, Shenzhen, China; ^2^Department of Sports Science and Physical Education, The Chinese University of Hong Kong, Shatin, Hong Kong, SAR China; ^3^School of Laboratory Medicine, Hubei University of Chinese Medicine, Wuhan, China

**Keywords:** physical activity, autism spectrum disorders, inclusive school, social-relational model of disability model, children

## Abstract

**Background:** The health benefits of physical activity (PA) participation are well-documented. Little was known about the PA levels of students with autism spectrum disorder (ASD) and their typically developing (TD) peers in inclusive schools. This study aimed to synthesize available studies examining PA levels of children and adolescents with and without ASD and its associated factors that affected their PA participation during inclusive schools applying the social–relational model of disability (SRMD).

**Methods:** Eight databases were searched including CINAHL Complete, SPORTDiscus with Full Text, PubMed, Embase, Web of Science, Eric, APA PsycINFO, and Scopus from inception through May 2021 to identify related studies. Two researchers independently screened studies, assessed methodological quality, and summarized relevant data. The McMaster Critical Reviewer Form for quantitative studies was used to evaluate the methodological quality of the included articles.

**Results:** A total of seven articles were included in this systematic review. Overall, meta-analysis results indicated that children and adolescents with ASD had a moderately decreased PA levels compared with their TD peers [SMD = −0.585, 95% CI (−0.774, −0.425), *p* < 0.01]. Individual-, social-, and environmental-level factors that influence PA levels in children and adolescents with ASD were identified from the perspective of SRMD.

**Conclusion:** This review indicates that children and adolescents with ASD have lower PA levels than their TD peers in inclusive schools and multilevel factors affect their PA.

## Introduction

Individuals with autism spectrum disorders (ASD) are dramatically characterized by deficient social communication, stereotyped behaviors, and intelligence development (1). The current prevalence of ASD in the global population is around 0.7–1.1% (2). Children and adolescents with ASD are of great sensitivity to changes in their environment and are prone to depend on routines (1). This may expose them to the limited opportunities to participate in exercise and physical activities (3). Additionally, a wide range of precipitating factors, including increased screen-based time, social skill impairments, and motor skill deficiencies, are likely to result in physical inactivity and sedentary behavior (4–6). Previous studies have indicated that children with ASD exhibit high rates of overweight and obesity (7). The consequences of physical inactivity could lead to a diversity of chronic diseases, such as obesity, diabetes, hypertension, cardiovascular disease, and mental disabilities (8).

Schools are valuable setting to engage students with and without disabilities to increase PA and shape their PA behaviors (9, 10). In light of this, examining the time slots contributing to the most PA participation during a school day is essential to better understand their PA patterns and to promote PA behaviors among students with ASD. Schools are identified as the best place to promote PA opportunities for all students according to the latest *WHO Guidelines on physical activity and sedentary behavior* (11); it recommends that children and adolescents with living disabilities aged 5–17 years should engage in at least 60-min moderate-to-vigorous physical activity (MVPA) daily to achieve health benefits. Achieving the recommended amounts of PA plays a crucial role to promote and maintain a life-long healthy active lifestyle for people of all ages and abilities. However, only 42% of children and adolescents aged 5–17 years with ASD met the WHO Guidelines (12). Schools are recommended to offer physical education (PE) class and recess period to enable students to gain MVPA opportunities. Especially, an increasing number of students with ASD have received their education in inclusive schools with their typically developing (TD) peers, with the implementation of legislation and policies regarding inclusive education (e.g., the Salamanca Statement). Inclusive education is an approach that aims to eliminate social exclusion on the premise that education is a foundation for society, and this concept has been accepted as a core education policy worldwide (13). A PA guideline (PAG) stated that children and adolescents should spend at least 50% of the PE class time and 40% of the recess period engaging in MVPA (14). The availability of PE classes and recess periods has been found to be effective in increasing PA in students with ASD (11, 12). Studies over the past two decades have provided important information on PA levels in children with ASD during school time in inclusive settings (e.g., inclusive PE class or inclusive recess). To date, limited attention has been emphasized to compare the PA levels of children and adolescents with and without ASD during a school day and take school PAG into consideration, especially for those who enrolled in the inclusive schools.

One previous systematic review summarized the objectively measured MVPA level on weekdays and weekends among children and adolescents with ASD (12). However, this review conducted an overview of MVPA of children and adolescents with ASD in diverse settings (e.g., special schools, home schools, and inclusive schools) as a whole, without a comparison with their TD peers. Therefore, it is difficult to identify the PA levels of students with and without ASD in inclusive schools, which cause barriers to design and implement effective interventions targeting inclusive school settings. In addition, the reasons for low PA levels in children and adolescents with ASD at school are complex. In the light of the nature of the ASD symptoms, students with ASD may meet various obstacles in PA engagement with their TD peers. Different factors ranging from personal, social, cultural, and environmental perspectives act as either facilitators or inhibitors for their PA participation (15). Therefore, there is a pressing need to develop a comprehensive review to determine the PA levels of children and adolescents with ASD compared with their TD peers, and to identify the factors that affect their PA participation in the setting of inclusive schools.

Because of the complexity of diverse factors affecting PA engagement, it is necessary to summarize multiple factors through a conceptual framework. The social-relational model of disability (SMRD) has been applied in previous studies to examine the individual, social, and environmental level of barriers to PA for individuals with disabilities (16). SMRD emphasized the social influence imposed on impairment by individuals without disabilities either through “barriers to doing” or “barriers to being” (17). Considering that the present study was based on inclusive setting, SMRD is a more appropriate theoretical framework than other models (e.g., social-ecological model) due to its underlying alignment to the morality of inclusion, in which personal impairment interacts with the social environments (18, 19). Therefore, this model was adopted to address the factors that inhibit or promote PA participation among children and adolescents with ASD in inclusive schools.

To the best of our understanding, no systematic reviews have yet examined the accelerometer-measured PA levels of children and adolescents with and without ASD in inclusive schools, and the associated factors that affect their PA levels applying a theoretical framework. The aim of this systematic review was two-fold. The first was to systematically review and quantitatively synthesize the published literature to determine the PA levels in ASD compared with TD children and adolescents in inclusive schools. The second was to identify the factors that affected the PA levels in children and adolescents with and without ASD at different levels using the SMRD as a theoretical framework.

## Methods

This study complied with the Preferred Reporting Items for Systematic Review and Meta-analyses Statement (PRISMA) (20).

### Search Strategy

Electronic searches were conducted in CINAHL Complete (via EBSCOhost), SPORTDiscus with Full Text (via EBSCOhost), PubMed, Embase (via Ovid), Web of Science, Eric (via EBSCOhost), APA PsycINFO (via Ovid), and Scopus from inception through May 2021 to identify all relevant published articles regarding the objectively measured PA levels and correlates in children and adolescents with and without ASD. The search was limited to “English,” “human-related,” and “peer-reviewed” articles if applicable to that database. The initial search was undertaken using the following key terms: physical activity, physical activity levels, ASD, children, or adolescents. The search keywords for each main term were developed from the search strategies of previous reviews related to PA and children or adolescents with ASD and expert opinions in the fields of PA and special education. To expand our search, a manual search in reference lists of retrieved articles and Google Scholar was also screened to identify relevant articles.

### Inclusion and Exclusion Criteria

Inclusion criteria were as follows: (a) objectively measured the PA levels (e.g., MVPA) of children and adolescents with ASD and compared with their TD peers; (b) observational research (i.e., cross-sectional, case-control, and cohort); (c) reported the PA levels in the form of duration in minutes in different inclusive school settings (e.g., inclusive physical education class, recess, lunchtime, and after-school time); (d) peer-reviewed articles with full-text available written in English; (e) included participants who are aged 5–17 years old; (f) provided complete research data where the length of MVPA could be computed. Exclusion criteria were as follows: (a) did not collect data from inclusive school settings; (b) only included participants with ASD without their TD peers; (c) written in a language other than English; (d) intervention research (e.g., clinical and field trials); (e) review studies, case/government reports, conference papers, book chapters, and policy documents; and (f) included preschool children (aged 0–5 years) and adult people up to 18 years old as subjects.

### Data Selection and Data Extraction

Two independent reviewers examined each article obtained from the databases to ensure the accuracy of the systematic search process. If two independent reviewers had any disagreement, the third reviewer would discuss dubious papers with the two reviewers and made a final call. The consistency of the title/abstract and full-text screening between the two reviewers was measured using the kappa value (21). A standardized data extraction form was developed to extract characteristics from each study, including the relevant data about bibliographic details (author and year), participant characteristics (sample size, age range, gender, school placement, location, and classification of ASD severity), outcome measures (measurement tools of PA), PAG cited in the study, study purpose, major findings, and PA-related factors applying in SMRD.

### Quality Assessment

The McMaster Critical Reviewer Form for quantitative studies (16) was used to evaluate the methodological quality of the included articles on the basis of the Guidelines for Critical Review Form-Quantitative Studies (22). The numerical rating criteria for non-experimental quantitative study developed by Imms (23) was also employed to interpret the methodological quality. These scoring criteria have been widely used in previous systematic reviews related to disability and PA research (12, 24, 25). The three key criteria in the included studies were evaluated in the present study: sample, measurement, and analyses (23). The sample was evaluated whether the selection bias was reduced (e.g., representative of selected population or convenience sample), whether the sample size was suitable for the research design and questions, and whether the characteristics of the subjects were clearly described by the authors. The measurement examined whether the measurement bias was reduced (e.g., reliability and validity of the measurement tool, recall/memory). The analyses examined whether reported analyses were appropriate for the research questions and outcome measures (e.g., reported statistical significance, point estimates, provided variability, and discussed clinical importance) (12, 24, 25). Each criterion was scored with one star, which means no evidence shows that the study can meet any criterion. Two stars indicate that certain pieces of evidence in the study can meet the criteria, or the report is unclear. Finally, three stars indicate that the evidence in the study can totally meet the criteria (23, 24). Two reviewers independently evaluated the methodological quality assessment for the included studies. Discrepancies between the two reviewers were discussed until consensus was finally reached. If an agreement could not be obtained from the two reviewers, then a third research could make the final call after discussing with two reviewers.

### Data Analysis

A meta-analysis was developed by the Comprehensive Meta-Analysis (v.2). The MVPA of children and adolescents with ASD and their TD peers was calculated to actual minutes during an inclusive school day including five settings (e.g., overall, PE class, recess, lunch time, and after-school time) to determine PA levels and calculate the effect size. Hedges' *g* was chosen as the effect size index as it was suitable for the included smaller number of studies (*k* < 20) (26). Data for each study were expressed as the standardized mean differences (*g*) using the random effects model and given weighted by the study inverse variance. The magnitude of Hedges' *g* values was interpreted as small (<0.2), moderate (0.5), and large (>0.8) effect size, respectively (27). To check for the existence of publication bias, funnel plots was presented. The *I*^2^ value was used to assess the heterogeneity of the effect sizes, indicating small (≤25%), medium (50%), and large (≥75%) amount of heterogeneity, respectively (28). Fifty percent of *I*^2^ value was set as a cutoff point to evaluate the heterogeneity of the included studies. If the value of *I*^2^ statistics was >50%, indicating an existence of the heterogeneity. In order to address heterogeneity, a sensitivity analysis was used to estimate potential outliers. Since <10 studies were included in each analysis, publication bias was not investigated. Statistical significance (*p* < 0.05) was set for all tests.

To identify factors as being “related” or “not related” to PA participation of students with ASD in inclusive schools, those potential factors showing significant association with PA participation were summarized.

## Results

### Study Identification

**Figure 1:**
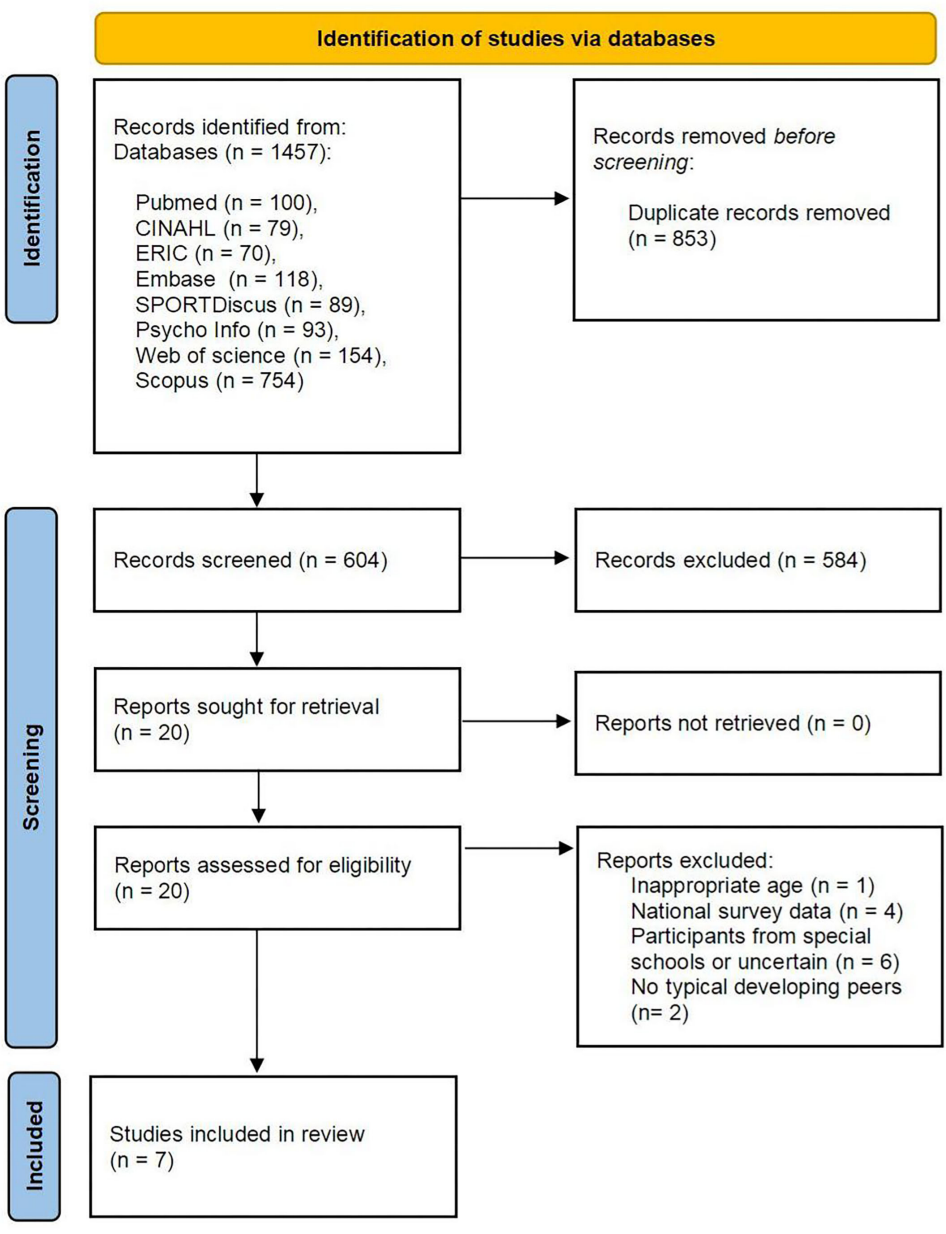
Flow diagram showing the study selection process (20).

A total of 1,457 articles were found in the initial search of the eight databases described. Figure 1 illustrates the procedure of screening and the number of articles that met the inclusion criteria. After removing the duplicates, 604 articles were further screened through title/abstract. Twenty of them potentially met the inclusion criteria with an inter-rater reliability kappa value = 0.84 between the two reviewers. Subsequently, full-text screening was conducted to identify eligible studies. Finally, seven articles were included in this systematic review and meta-analysis, with an inter-rater reliability of kappa value = 0.94.

### Descriptive Characteristics of Included Studies

The characteristics of included studies are summarized in Table 1. All included studies used a cross-sectional design, and only one study was conducted in the USA (14%) (29), whereas 86% of the included studies were from Taiwan (3, 30, 31, 33–35). The total sample included 172 children and adolescents with ASD and 277 of their TD peers aged from 9 to 15 years, and 97% of the participants were male students. The school placement of participants in the included studies ranged from primary school to high school: primary school (43%) (29–31), secondary schools (43%) (33–35), and high school (14%) (3). Six included studies (86%) provided a clear classification of ASD severity (29–31, 33–35). All included studies used accelerometers as an objective measuring instrument to assess the PA levels of children and adolescents with ASD and their TD peers. Only Sandt and Frey (29) adopted both accelerometer and direct observation using BEACHAES observation form to measure PA levels of students with and without ASD. The included studies cited different PAG as outcome measures to evaluate the number of participants and amount of time that meet the standard PAG.

**Table 1:** Summary of the participants' characteristics of included studies.

	**Sample size**	**Gender (ASD; TD)**	**Age range (mean, SD)**	**School level**	**Location**	**Classification of ASD severity**	**Measures of PA**	**PAG**	**Quality criteria**		
									**Sample**	**Methods**	**Analysis**
Sandt and Frey (29)	15 ASD; 13 TD	10M, 5F; 8M, 5F	5–12 (9.5, 1.9); 5–12 (8.9, 2.0)	PS	USA	Autism (9), Asperger syndrome (2), PDDNOS (4)	Accelerometer & Observation (BEACHES)	a	^**^	^***^	^***^
Pan (30)	24 ASD; 24 TD	23M, 1F; ?, ?	7–12 (?, ?); 7–12 (9.2, 1.4)	PS	Taiwan	Autism (21), Asperger syndrome (3)	Accelerometer	b, c	^**^	^**^	^***^
Pan (31)	24 ASD; 24 TD	23M, 1F; 23M, 1F	7–12 (9.3, 0.87); 7–12 (9.13, 0.68)	PS	Taiwan	Mild or high-functioning autism (12), moderate autism (9); Asperger syndrome (3)	Accelerometer	c	^**^	^**^	^***^
Pan et al. (3)	19 ASD; 76 TD	19M; ?, ?	? (14.19, 0.82); ? (14.1, 0.80)	HS	Taiwan	NR	Accelerometer	b	^*^	^**^	^*^
Pan et al. (32)	25 ASD; 75 TD	25M; 75M	? (14.26, 0.89); ? (14.08, 0.80)	SS	Taiwan	Mild autism (15), Asperger syndrome (10)	Accelerometer	b	^**^	^**^	^**^
Pan et al. (33)	30 ASD; 30 TD	30M; 30M	12–17 (14.51, 1.54); 12–17 (14.72, 1.54)	SS	Taiwan	Mild autism (23), Asperger syndrome (7)	Accelerometer	a, b, c	^**^	^**^	^**^
Pan et al. (34)	35 ASD; 35 TD	35M; 35M	12–17 (14.55, 1.54); 12–17 (14.81, 1.55)	SS	Taiwan	Mild autism (25), Asperger syndrome (10)	Accelerometer	a	^**^	^*^	^***^

*M, male; F, female; TD, typical development; SS, secondary school; HS, high school; PS, primary school; SD, standard deviation; NR, not reported; PDDNOS, Pervasive Developmental Disorder—Not Otherwise Specified; ?, no data provided; PAG, physical activity guideline; BEACHES, Behaviors of Eating and Activity for Children's Health: Evaluation System; a, children and adolescents should spend at least 60 min in MVPA daily; b, children and adolescents should have 50% of PE class time in MVPA; c, children and adolescents should have 40% of recess time in MVPA*.

* *No criteria was met within that component*.

** *Only some criteria were met within component*.

*** *All criteria were met within that component*.

#### Meta-Analysis of Time Spent in Moderate-to-Vigorous Physical Activity at Different Settings

**Figure 2:**
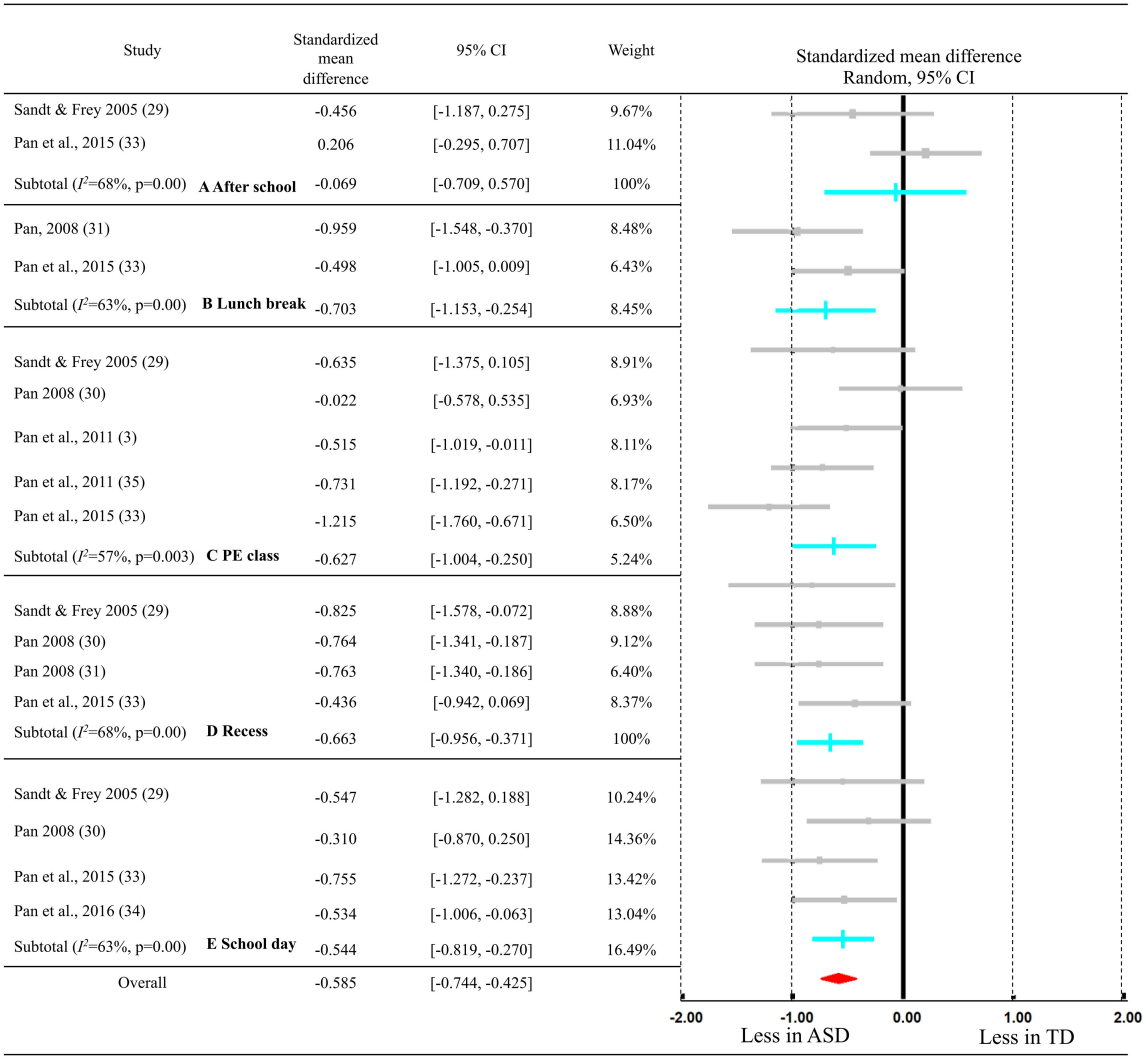
Meta-analysis of difference of the time spent in MVPA between children with ASD and TD after school **(A)**, during lunch break **(B)**, at PE class **(C)**, at recess **(D)**, and during the school day **(E)**.

Finally, seven studies including data of 172 ASD populations and 277 of their PD peers were included in the meta-analysis. The results of the meta-analysis are shown in the Figure 2. It indicated the overall effect size for overall PA levels and separated by specific school settings. Overall, the meta-analysis indicates that children with ASD had moderately decreased PA levels compared with TD [SMD = −0.585, 95% CI (−0.774, −0.425), *p* < 0.01], with small-to-medium heterogeneity (*Q* = 23.614, *I*^2^ = 32%, *p* = 0.099). Specifically, two studies focusing on after-school time (see Figure 2A, after school) and reported that children with ASD (mean = 38.07 min) had non-significant small decreased PA levels compared with TD (mean = 41.42) [SMD = −0.069, 95% CI (−0.709, 0.570), *p* = 0.832], with a medium heterogeneity (*Q* = 2.145, *I*^2^ = 53%, *p* = 0.143). For the lunch break (see Figure 2B, lunch break), two studies focused on this time periods and reported that children with ASD (mean = 7.49 min) had a moderate to large significant decreased PA levels compared with TD (mean = 10.57 min) [SMD = −0.703, 95% CI (−1.153, −0.254), *p* = 0.002], with a small to moderate heterogeneity (*Q* = 1.352, *I*^2^ = 26%, *p* = 0.245). The meta-analysis of MVPA during PE class (see Figure 2C, PE class) showed a significant and moderate decrease in ASD (mean = 14.90 min) compared with TD (mean = 19.07 min) children [SMD = −0.627, 95% CI (−1.004, −0.250), *p* = 0.001], with a medium heterogeneity (Q = 9.406, *I*^2^ = 57%, *p* = 0.052). The MVPA during recess time (see Figure 2D, recess) also indicated a moderate effect with a lower score in ASD (mean = 24.72 min) compared with TD (32.68 min) children [SMD = −0.663, 95% CI (−0.956, −0.371), *p* < 0.00], with a small heterogeneity (Q = 1.182, *I*^2^ = 0%, *p* = 0.757). Last, for overall MVPA during a school day (see Figure 2E, school day), four studies reported that children and adolescents with ASD (mean = 69.51 min) had a significant and moderate decrease in PA levels compared with TD (mean = 93.97 min) [SMD = −0.544, 95% CI (−0.819, −0.270), *p* < 0.00], with a small heterogeneity (*Q* = 1.306, *I*^2^ = 0%, *p* = 0.728).

#### Factors Affecting the Physical Activity Levels of Children and Adolescents With Autism Spectrum Disorder

Three-level factors that affect the PA levels of children and adolescents with ASD in inclusive school settings have been identified by previous researchers and summarized from included studies in Table 2. At the individual level, age is a crucial factor that affect the PA levels of children and adolescents with ASD (30). In addition, the sedentary pursuits, physical fitness level, self-determined motivation, and social impairment play a key role in determining the PA levels of children and adolescents with ASD (3, 29–31, 33, 34). At the social level, PE class should be the focus. PE content, behaviors and social interactions of PE teachers with TD peers during PE class affected the PA levels of students with ASD during their inclusive PE (3, 29–31, 33). Last, at the environmental level, in-school PA opportunities (e.g., PE, recess, lunch time, and after-school PA programs) and school environment (playground, PA equipment, and facilities) have great influences on PA levels of children and adolescents with ASD during school days.

**Table 2:** Summary of included studies on physical activity (PA)-related findings in inclusive schools.

**References**	**Study design**	**Study purpose**	**Major findings**	**PA-related factors of students with ASD in SRMD**		
				**Individual**	**Social**	**Environment**
Sandt and Frey (29)	Cross-sectional	Compare PA levels and patterns between children with and without ASD	Children with ASD were similarly active in recess and PE than children without ASD	Sedentary pursuits (technology-based activities) 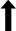	Unstructured after-school activities 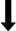 ; excessive class management and unmodified instructions by PE teachers 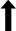 ; lack of APE specialists 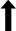	Limited recess time 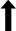
Pan (30)	Cross-sectional	Compare MVPA of students with ASD and TD students during inclusive PE and recess	Students with and without ASD spent a larger percentage of time in MVPA during PE compared with recess period	Social impairment (lack of verbal or physical prompts during inclusive recess) 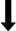	PE teacher support (demonstration and physical assistance) 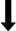 ; PE content adjustment (isolated context) 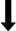 ; PE content and location (fitness and outdoor) 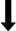	School environment (limited spaces, equipment, and playground facilities) 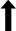
Pan (31)	Cross-sectional	Compare the PA levels of children with ASD and TD children during inclusive recess settings	Children with ASD were less active during overall recess compared with their TD peers	Age (old children to be active in recess) 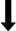 ; sedentary pursuits (directly go home after school) 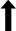	Lack of support and instruction during recess 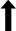 ; teachers' behaviors (curricula accommodation and assignments) 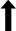	NR
Pan et al. (3)	Cross-sectional	Examine PA behaviors and correlates that may affect the PA of adolescents with and without ASD during inclusive PE	Adolescents with ASD were less physically active than their TD peers, their PA was related positively to their social interaction with TD peers	NR	PE content and location (fitness test, free-play, and outdoor) 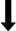 ; teacher-related characteristics (female teachers and non-certified teachers) 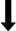 ; social initiations and interactions with TD peers 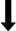	NR
Pan et al. (35)	Cross-sectional	Examine PA and motivation between adolescents with and without ASD during inclusive PE	Adolescents with ASD had less PA levels in PE and lower motivations toward PE than adolescents without ASD	Motivation (external regulation) 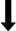 ; age 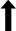 ; social impairment (less self-motivated) 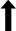	Teacher support (providing feedback and encouragement) 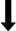	NR
Pan et al. (33)	Cross-sectional	Compare the PA intensity of secondary school-aged students with and without ASD during a school day	Students with ASD had significantly lower daily PA than TD students during a school day	Age (PA declines with age) 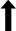	PE teachers lack APE training 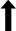 ;	In-school PA opportunities (PE, recess, and lunchtime) 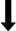
Pan et al. (34)	Cross-sectional	Compare PA and physical fitness between secondary school-aged male students with and without ASD	Students with ASD were less physically active overall and had significantly lower scores on physical fitness measures than their TD peers	Fitness level (cardiovascular endurance, muscular strength, and endurance) 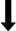	Lack of extracurricular PA programs 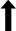	NR

*NR, not reported; MVPA, moderate to vigorous physical activity; 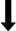, positive association; 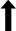, negative association; ASD, autism spectrum disorder; TD, typically developing*.

#### Quality Assessment

Assessment of the methodological quality of included studies according to the McMaster Critical Reviewer Form is reported in Table 1. None of the studies met all the three criteria. For the *Sample* component, all studies used a convenience sample, and male participants dominated the sample selection. Six studies gave detailed classification of ASD diagnosis. For the *Measurement* component, only one study was given three stars as adopting both objective tool and observation form to record PA levels. For the *Analyses* component, four studies were given three stars because they fully addressed the research questions and clearly explained the limitations.

## Discussion

The aim of this systematic review and meta-analysis was to determine the PA levels of children and adolescents with and without ASD in inclusive schools and identify the PA-related factors at three levels that affect PA engagement of children and adolescents with ASD in inclusive schools. In general, the results showed evidence for lower PA levels in ASD compared with TD children. A previous systematic review (36) found that individuals with ASD (aged 0–18 years old) engaged in approximately 86 min in of MVPA daily (ranging from 34 to 188 min/day). Our synchronized results of time in MVPA in inclusive schools was 69.51 min/day, which fell within this range. Comparatively, a recent study indicated that TD students spent around 135 min in MVPA daily (37), and our calculated results (101.96 min/day) for TD peers in inclusive schools is greatly lower than their results. A recent study in children with ASD in special schools in Spanish found that children with ASD recorded approximately 70 min of MVPA during weekdays (38), and their measured results in special schools were slightly lower than our calculated results in inclusive schools. In addition, a previous study in 13 special schools in Hong Kong recruiting 259 children with five types of disabilities including children with ASD found that children spent 70% of their school time being sedentary and only acquired 17 min in MVPA (10). Therefore, the results may suggest that children and adolescents with ASD who attended inclusive schools were more likely to have opportunities to accrue more MVPA daily than those with ASD attending special schools. Specifically, students with ASD spent less time in MVPA than non-ASD peers during PE lessons (37.3 vs. 47.3%, SMD = −0.627). Although students without ASD cannot meet the PE lessons criteria in inclusive schools, they acquired more MVPA than elementary school-aged TD students (44.8%) (39) and students in secondary school PE lessons (40.5%) (40). In addition, students with ASD consumed less time in MVPA than their TD peers (31 vs. 40.1%, SMD = −0.663). The PA intensity achieved by students with ASD during inclusive recess is higher than students with physical disabilities in special schools in Hong Kong (17%) (41), 22% of special school population in Hong Kong (9.4%) (10), and adolescents with intellectual disabilities in inclusive recess in Taiwan (17.89%) (42), but they are less active than primary school students with intellectual disabilities in the USA (78.3%) (43). Furthermore, during lunch break, students with ASD achieved lower levels of PA than their TD peers (16.3 vs. 22.2%, SMD = −0.703), but the PA levels of students with ASD during lunch time in inclusive schools is greatly higher than students with special education needs (SEN) in special schools in Hong Kong (4.5%) (10), and students with physical disabilities in Hong Kong (14.1%) (41). Based on the latest *WHO Guidelines on physical activity and sedentary behavior* (11), schools should provide tailor-made programs in specific settings (e.g., PE and recess) to promote MVPA for children and adolescents with ASD to meet the daily 60 min of MPVA guidelines.

The accelerometer has been widely used to measure PA for children and adolescents with and without ASD as an objective measuring tool in inclusive school. In addition, only one study (29) adopted the accelerometer and the Behaviors of Eating and Activity for Children's Health: Evaluation System (BEACHES) to collect PA data. It is better to utilize two different types of PA measuring tools to collect PA data for children and adolescents with disabilities (44). It is still necessary to notice that none included studies using questionnaires or interviews to gather richer data from educational stakeholders such as parents, teachers, TD peers, and students with ASD. Using the accelerometer alone can only get the quantified data. As to why students with ASD cannot fully participate in PA, further observations, questionnaires, and interviews for diverse educational stakeholders can help researchers know more details.

The reasons for low PA levels in children and adolescents with ASD are complex. In compliance with the SRMD, the factors affecting PA levels and PA participation can be divided into three levels ranging from individual to environmental level.

At the individual level, in general, a decline in PA with age is recorded, especially in recess time. Primary-school-aged students with ASD spent 31.38% of recess time in MVPA, whereas secondary-school-aged students with ASD only spent 21.24% of recess time in MVPA. A previous review also echoed this finding that age was consistently inversely related to PA in children with ASD (36). One possible explanation is that as children become older, game rules and required motor skills become more complex. Students with ASD cannot adopt in competitive group games with TD peers and always selected low intensity and solo games during recess time (45). Sedentary pursuits, such as playing with technology-based activities during after-school time, have negative effects on children and adolescents with ASD to shape active behaviors. One systematic review calculated that children with ASD spent an average of 479 min/day in sedentary behaviors including an average of 271 min/day in screen time (36). Another study also reported that children with ASD spent over 62% more time on screen-based activities (e.g., TV viewing and video games) (46). Long engagement with electronic screen activities during after-school time at home has been reported by parents of children with ASD and children with ASD themselves as the most common reason for decreased PA levels (15, 47). Only one study focused on the associations between motivation and PA levels in adolescents with ASD and found that external regulation was positively and significantly related to the time spent in MVPA during the inclusive PE class (35). One possible explanation is that adolescents with ASD were afraid of being isolated by their friends and TD peers (external regulation) during the inclusive PE class. However, it is noticed that PA participation can be regarded as an external award for children with ASD, and fear of isolation by TD peers (introjected regulation) as their motivation to be active during inclusive PE may be not beneficial for their mental health. Therefore, there is a need to develop their intrinsic motivation to actively participate in inclusive PE with their TD peers. Social impairment of students with ASD also decreased their PA levels especially in inclusive recess. One previous review also reported that higher levels of encouragement from friends were related to higher PA levels during recess time (48). However, for adolescents with ASD, they frequently reported that they were socially isolated and preferred solitary activities (49). Therefore, students with ASD hardly participate in PA programs with TD peers during unstructured time.

At the social level, PE lessons were reported to be the focus. PE teachers reported several barriers to implement inclusive PE, such as lack of APE specialists, professional knowledge, and training regarding teaching students with ASD in general PE lessons and modification of PE content and instruction (29, 30, 33). These barriers are commonly reported by inclusive PE teachers and need support by the school (50, 51). In addition, tailored PE content and appropriate equipment and facility location are positively associated with MVPA of students with ASD (12). Well-designed PE lessons not only can help students with disabilities grasp opportunities to accumulate MVPA daily but also shape their PA behaviors as a primary institution (9, 10, 52). Last, schools cannot organize enough after-school PA programs to promote PA participation for all students. Organized extracurriculum PA programs in schools can accept children with ASD to accumulate MVPA daily, decrease sedentary time, and improve social interaction skills with TD peers.

At the environmental level, very little research recorded school environment and examined the effects of school environment on the PA levels of students with ASD during inclusive schools. Only one study found that limited space, equipment, and playground restricted students with ASD from acquiring enough MVPA (31). Previous studies have reported that teachers lacked equipment and facilities to include students with SEN in general PE (53, 54). In addition, it is worth noting that in-school PA opportunities are very different. Some schools provided limited recess and lunch time and moved students with ASD quickly to the classroom to prepare for the next course after lunch (29–31). Thus, schools are much needed to formulate written policies to regulate recess and lunch time at school level because a clear and mandatory policy for recess and lunch time cannot only promote PA engagement of students with and without SEN but also help them maintain health and improve the quality of life into adulthood.

The limitations of this review are noteworthy. First, the total number of included studies are limited, which caused bias in summarizing the results. Second, all included papers have utilized convenience sampling method to recruit participants, which can cause a high level of sampling bias and reflect the pointed PA levels of individuals with ASD. We also noticed a heavy gender difference; only 3% of the participants are females so that we cannot examine whether gender was associated with PA levels in this population. Third, only the studies that indicated detailed minutes spent for MVPA in inclusive schools and compared PA levels with TD students are included. This inclusion criterion may miss some studies and cause bias in determining the PA levels of children and adolescents with ASD. Fourth, the majority of included studies only used objective measurement to calculate PA levels of children with ASD so that there is lack of details to understand PA acquisition from the subjective perspectives. Last, external validity may be low. This review is limited by insufficient participants and geographic differences (six studies in Taiwan and one study in the USA), indicating that the results cannot be widely applied.

## Conclusion

In sum, the PA levels of children and adolescents with ASD are relatively lower than those of their TD peers in an inclusive school. Limited studies have been developed to focus on children and adolescents with ASD, especially those who attended inclusive schools. Based on this review, in-school PA intervention programs are much needed to design the promotion of PA levels of children and adolescents with ASD during school days. Future studies also are encouraged to explore more diverse variables (e.g., gender, educational stakeholders, school environment, policies, etc.) to identify the effects of those variables on the PA levels in children and adolescents with ASD in inclusive schools.

## Data Availability Statement

The original contributions presented in the study are included in the article/Supplementary Material, further inquiries can be directed to the corresponding authors.

## Author Contributions

XL and ZR contributed to the design of this study. RL and XL acquired the data and drafted the manuscript. RL, XL, and YZ interpreted the data and performed the statistical analysis. All authors contributed to the revision and approval of the submitted and final version of this manuscript.

## Funding

This work was supported by the Philosophy and Social Science Program of Guangdong Province (No. GD19YTY02) and High-level Scientific Research Foundation for the Introduction of Talent of Shenzhen University (grant number RC00228). The funder had no involvement in the design of the study, analysis and interpretation of the data, decision to publish, or preparation of the manuscript.

## Conflict of Interest

The authors declare that the research was conducted in the absence of any commercial or financial relationships that could be construed as a potential conflict of interest.

## Publisher's Note

All claims expressed in this article are solely those of the authors and do not necessarily represent those of their affiliated organizations, or those of the publisher, the editors and the reviewers. Any product that may be evaluated in this article, or claim that may be made by its manufacturer, is not guaranteed or endorsed by the publisher.

## Abbreviations

PA: physical activity
ASD: autism spectrum disorder
TD: typically developing
SRMD: social–relational model of disability
MVPA: moderate to vigorous physical activity
PE: physical education
PAG: physical activity guideline
SEN: special education needs.
